# Telomere-to-Telomere Genome Sequences across a Single Genus Reveal Highly Variable Chromosome Rearrangement Rates but Absolute Stasis of Chromosome Number

**DOI:** 10.3390/jof8070670

**Published:** 2022-06-25

**Authors:** Mathieu Quenu, Artemis D. Treindl, Kate Lee, Daigo Takemoto, Torsten Thünen, Samad Ashrafi, David Winter, Austen R. D. Ganley, Adrian Leuchtmann, Carolyn A. Young, Murray P. Cox

**Affiliations:** 1 School of Natural Sciences, Massey University, Palmerston North 4442, New Zealand; kate.d.lee@gmail.com (K.L.); david.winter@esr.cri.nz (D.W.); 2 Plant Ecological Genetics, Institute of Integrative Biology, ETH Zürich, 8092 Zürich, Switzerland; artemis.treindl@wsl.ch (A.D.T.); adrian.leuchtmann@retired.ethz.ch (A.L.); 3 Graduate School of Bioagricultural Sciences, Nagoya University, Nagoya 464-8601, Japan; dtakemo@agr.nagoya-u.ac.jp; 4 Julius Kühn Institute–Federal Research Centre for Cultivated Plants, Institute for Crop and Soil Science, 38116 Braunschweig, Germany; torsten.thuenen@julius-kuehn.de; 5 Julius Kühn Institute–Federal Research Centre for Cultivated Plants, Institute for Epidemiology and Pathogen Diagnostics, 38116 Braunschweig, Germany; samad.ashrafi@julius-kuehn.de; 6 School of Biological Sciences, University of Auckland, Auckland 1010, New Zealand; a.ganley@auckland.ac.nz; 7 Digital Life Institute, University of Auckland, Auckland 1010, New Zealand; 8 Noble Research Institute, Ardmore, OK 73401, USA; cayoung.kiwi@gmail.com

**Keywords:** *Epichloë*, genome structure, chromosome evolution, structural variation

## Abstract

Genome rearrangements in filamentous fungi are prevalent but little is known about the modalities of their evolution, in part because few complete genomes are available within a single genus. To address this, we have generated and compared 15 complete telomere-to-telomere genomes across the phylogeny of a single genus of filamentous fungi, *Epichloë*. We find that the striking distinction between gene-rich and repeat-rich regions previously reported for isolated species is ubiquitous across the *Epichloë* genus. We built a species phylogeny from single-copy gene orthologs to provide a comparative framing to study chromosome composition and structural change through evolutionary time. All *Epichloë* genomes have exactly seven nuclear chromosomes, but despite this conserved ploidy, analyses reveal low synteny and substantial rearrangement of gene content across the genus. These rearrangements are highly lineage-dependent, with most occurring over short evolutionary distances, with long periods of structural stasis. Quantification of chromosomal rearrangements shows they are uncorrelated with numbers of substitutions and evolutionary distances, suggesting that different modes of evolution are acting to create nucleotide and chromosome-scale changes.

## 1. Introduction

Despite the recent flood of genomic data, eukaryotic genomes assembled to chromosome-level remain uncommon [1] and the availability of multiple finished genomes from a single genus is extremely rare [2,3]. As a consequence, our understanding of how chromosome structure evolves through time is not well advanced [4]. We know that major structural changes are common, ranging from whole chromosome number variation (chromosome fission and fusion, aneuploidy, polyploidy, and accessory chromosomes) to smaller rearrangements within and between existing chromosomes [5]. On this smaller scale, sequence rearrangements are often shaped by forces including transposition, inversion and duplication [6], but the order and pace of these structural changes has not been studied in depth.

Within the eukaryotic kingdom Fungi, Saccharomycetales yeasts have been the most well studied, in large part due to their unusually small genome sizes [7], and yeast genomics has thus provided most insights into the evolution of structural genomic rearrangements [3,8]. However, the genomes of these unicellular yeasts are not strongly representative of most multi-cellular fungi lineages, particularly because they lack abundant transposable elements that are a feature of most eukaryote genomes. Finished genome assemblies of multiple species within a single genus of filamentous fungi are still uncommon. Comparisons of chromosome content in the genus *Verticillium* revealed considerable sequence rearrangements between species of the vascular plant pathogen [9], leading to low levels of chromosome conservation and aneuploidy within the genus. Whether this pattern extends to other genera and lineages of filamentous fungi is still unknown.

*Epichloë* is a genus of filamentous ascomycete fungi that are obligate symbionts of cool season grasses [10]. They have been studied extensively as a result of their important ecological roles as mutualists producing alkaloids that protect their grass hosts from herbivory [11]. The genus comprises a mix of haploid species generally able to reproduce sexually [12,13] and non-sexual hybrid species that do not cross back to parental lineages [14,15]. The genomes of *Epichloë* species sequenced to date are highly compartmentalized into distinct gene-rich regions adjacent to repeat-rich regions depleted in genes [16,17].

To facilitate a detailed understanding of chromosome evolution in genomes more representative of multi-celled eukaryotes, we have used long- and short-read sequencing to generate 15 complete telomere-to-telomere genomes from 12 *Epichloë* species, with 11 genomes newly presented here. These genomes comprise an evolutionary “transect”—a systematic sampling across the phylogenetic diversity of an entire genus. We focused on the non-hybrid haploid lineages so that our view of chromosome evolution is not confounded by hybridization. The taxonomic scope of this dataset permits a detailed analysis of chromosome change right across a genus, allowing us to identify the primary patterns and trends of chromosome evolution at different evolutionary distances, from the level of strains within a single species, through to deeply divergent lineages.

## 2. Materials and Methods

### 2.1. Genome Sequencing

Genomes were sequenced from high molecular weight genomic DNA obtained as described in Winter et al. [16]. Sequencing runs were performed either on the Oxford Nanopore MinION Mk1B using a R10.3 flow cell or the PacBio RSII or Sequel II instruments, depending on the sample (Table 1). Genomes were assembled using Canu v2.1.1 [18], with ambiguous breaks resolved using either Mecat2 v20193.14 [19] for PacBio data or Necat v0.0.1 [20] for Nanopore data. Genome polishing was performed with Pilon v1.24 [21], using Illumina short read libraries, where available (Table 1), or the Pilon long-read option. Homologous DNA lies across different chromosomes in *Epichloë*, therefore chromosomes were labelled by size within each strain, according to standard conventions.

### 2.2. Genome Annotation and Repeat Content Analysis

Gene models were called *de novo* for each species separately using the standard settings of funannotate v1.8.9 (https://funannotate.readthedocs.io; accessed on 1 March 2022). Repeat elements were identified using RepeatModeler v2.0.2a [22] and RepeatMasker v4.1.2.p1 (https://www.repeatmasker.org; accessed on 1 March 2022). Regions of low GC content, typically caused by Repeat-Induced Point (RIP) mutations in this genus, were identified with OcculterCut v1.1 [23]. Densities across the genome, as well as the fraction of repeat elements found in AT-rich regions, were calculated using the ‘coverage’ function of bedtools v2.30.0 [24].

### 2.3. Phylogenetic Analysis

Conserved gene orthologs were independently identified in each species with BUSCO v5.2.2 [25] using the closest reference database, hypocreales_odb10. Single copy ortholog protein sequences were passed to OrthoFinder v2.5.4 [26], which generated a maximum likelihood species phylogeny using RAxML-NG v1.0.3 [27] based on 1,489 orthologs with <5% missingness across species. Inclusion of two outgroup species with draft assemblies, *Claviceps paspali* C7990 (NCBI accession number GCA_000223175.2) [28] and *C. purpurea* C20.1 (GCA_000347355.1) [29], allowed rooting of the tree. Branch support values were calculated using the Shimodaira-Hasegawa algorithm in FastTree v2.1.10 with 1000× resampling.

### 2.4. Genome Synteny

Rearrangements of coding regions between chromosomes across different species were inferred and visualized using MCScan (python version) [30]. Fasta files containing DNA sequences of genes identified by the annotation pipeline were generated using the ‘getfasta’ function of bedtools v2.30.0 [24]. These sequence files and their corresponding annotation files were passed to the JCVI python module of MCScan, which built pairwise genome comparisons. For visual clarity, four species from major clades in the genus phylogeny (*E. gansuensis*, *E. glyceriae*, *E. baconii*, *E. typhina* 1756) were selected to illustrate rearrangements across all seven chromosomes.

### 2.5. Genome-Scale Multiple Alignment and Ancestral Genome Reconstruction

Genomes were aligned using Progressive Cactus v2.0.3 [31], a reference-free genome scale alignment program. The rooted phylogeny was used as a guide tree, and ancestral genome sequences were reconstructed at internal nodes in the tree. Mutations were counted from extant genomes to the sequence of the most proximal ancestor using the hierarchical alignment HAL toolkit v2.1 [32]. Eight classes of mutation were counted, corresponding to nucleotide substitutions (transitions, transversions), insertions/deletions of one or more nucleotides, and larger sequence rearrangements (inversions, duplications, transpositions). Linear regression models were built in R v4.1.0 [33] to check for correlations between mutation types and evolutionary distances, or between different mutation types. Probability values were calculated using Student’s *t*-test. The locations of structural variations on extant genomes were extracted via the halLiftover function of the HAL toolkit v2.1 [32] and compared to the location of AT-rich regions using bedtools v2.30.0 [24].

## 3. Results

### 3.1. Telomere-to-Telomere Genome Sequences for 15 Epichloë Species and Strains

Genomes were sequenced, completely assembled and annotated for 15 strains from 12 *Epichloë* species (Table 1), representing 85% of the haploid non-hybrid species currently recognized in the genus. All major clades in the genus *Epichloë* are represented, such that the most recent common ancestor of all sampled species is equivalent to the root of the genus phylogeny. All genomes were assembled to chromosome level, and consist of one circular mitochondrial sequence and seven nuclear chromosomes, including a resolved rDNA locus. The number of annotated genes varies from 6,489 to 8,324 per genome (Table 2).

**Figure 1:**
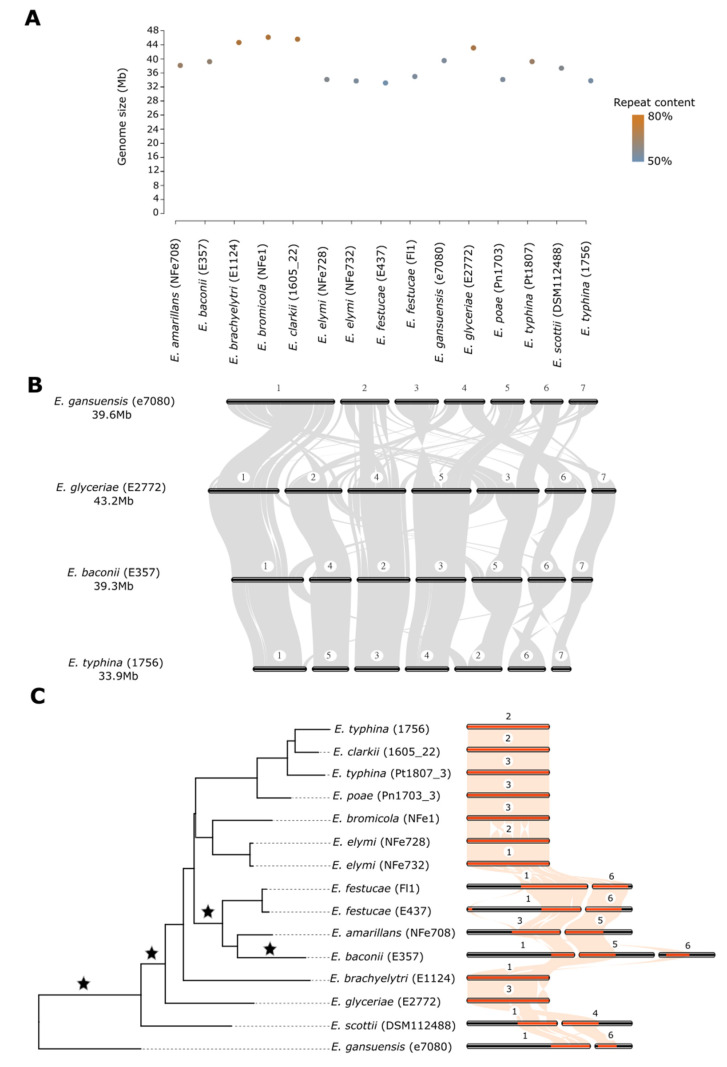
(**A**) Genome sizes for 15 *Epichloë* strains. The color of points indicates the repeat element content (%) of each genome. (**B**) Genome-wide rearrangements between four *Epichloë* species, inferred from gene-based syntenic analysis. Chromosomes are numbered relative to their size within each species, as the extensive rearrangements prevent homology-based numbering. (**C**) Evolutionary restructuring of a representative chromosome across the genus *Epichloë*. Sequences homologous in gene content to chromosome 2 in *E. clarkii* are highlighted in orange. Stars indicate positions in the phylogeny where major inter-chromosomal rearrangements have occurred. The chromosome numbers for each species are indicated.

**Figure 2:**
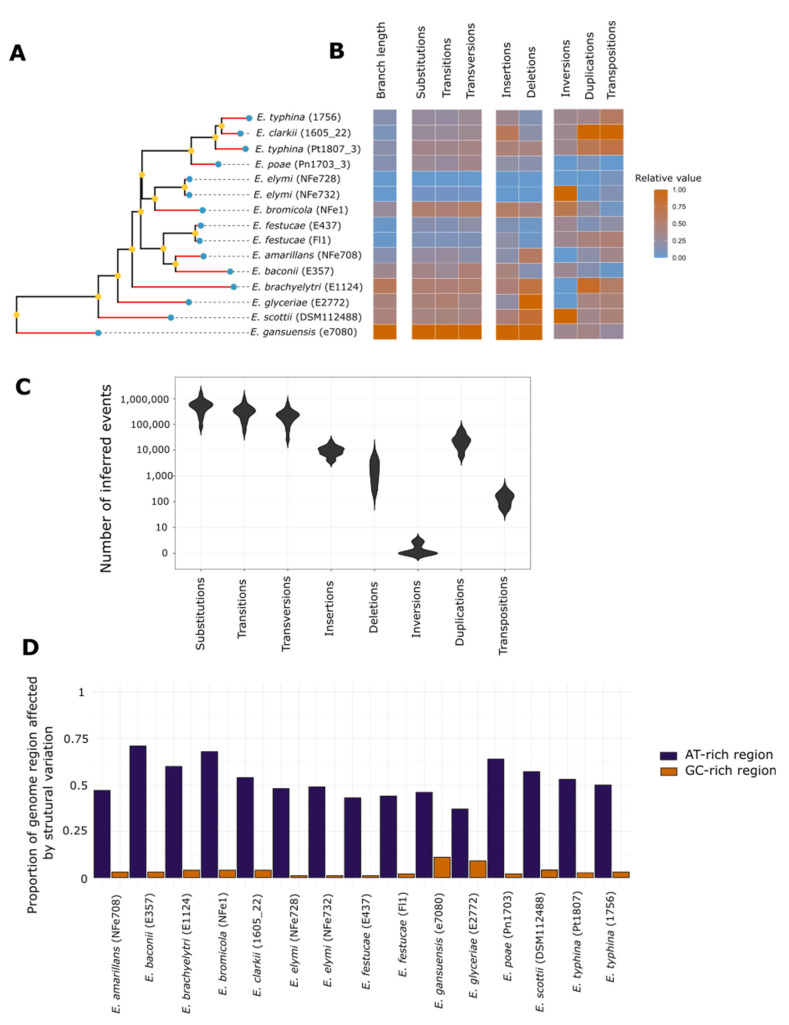
(**A**) Phylogenetic relationships between the 15 *Epichloë* species/strains inferred using maximum likelihood from 1,489 single copy gene orthologs. Blue dots indicate the positions of extant genomes; yellow dots indicate inferred ancestral genomes; mutations inferred by Progressive Cactus were counted on the red branches. All branches have support values of 1. (**B**) Heatmap highlighting the relative numbers of eight mutation classes accumulated on the terminal branches of the phylogeny between the closest ancestral genome and extant taxa. All rates are scaled from zero to one, with zero corresponding to the lowest number of mutations in the dataset and one to the highest. (**C**) Violin plots showing the absolute numbers of the eight mutation classes accumulated on terminal branches, with a log-scaled *y* axis. (**D**) Proportion of gene and repeat regions impacted by structural variation in each *Epichloë* strain, relative to the ancestral genomes inferred using Progressive Cactus. For each genome, the proportion of affected nucleotides was calculated by looking at the coverage of sequences duplicated, inverted, transposed and inserted relative to the closest ancestral genome.

We generated a species phylogeny using 1,489 single-copy gene orthologs (Figure 1C and Figure 2A), which recapitulates the known topology of *Epichloë* [34]. For instance, *E. clarkii*, *E. poae* and the two strains of *E. typhina* cluster in a monophyletic group that corresponds to the established *E. typhina* species complex; strains of *E. elymi* group with *E. bromicola*; strains of *E. festucae* group with *E. amarillans* and *E. baconii*; and *E. brachyelytri*, *E. glyceriae*, *E. scottii* and *E. gansuensis* are all positioned as basal single-species lineages. All branches in the phylogeny have high support values, so we consider that this phylogeny provides a robust evolutionary framework for interpreting changes in chromosome-level structure.

### 3.2. Large Genome Size Variation Is Primarily Due to Repeat Element Dynamics

A striking feature of the *Epichloë* genomes is that all have seven chromosomes. Despite this, the different species and strains exhibit large variation in genome size (Figure 1A, Table 2), ranging from 33.2 Mb (*E. festucae* E437) to 46.2 Mb (*E. bromicola* NFe1). They also exhibit variation in gene number (Table 2) that is uncorrelated with genome size (R^2^ = 0.02, *p* = 0.56). Instead, larger genomes consistently have higher repeat content (Table 2, positive correlation R^2^ = 0.81, *p* = 2.89 × 10^−6^), indicating that genome size is largely determined by insertion/deletion of repeats. Specifically, there was significant correlation between genome size and the two most abundant classes of retrotransposons, LTR-copia (R^2^ = 0.53, *p* = 0.003) and LTR-gypsy (R^2^ = 0.55, *p* = 0.002), suggesting a dominant role for these repeat classes in the genome size changes observed in *Epichloë*. Transposable elements in *Epichloë* are typically deactivated by Repeat-Induced Point (RIP) mutation, which induces cytosine to thymine transitions in any repeated genomic sequences [35]. All *Epichloë* genomes show striking compartmentalization, with distinct blocks of AT-rich and GC-rich regions [16,17]. Consistent with the AT-rich regions deriving from RIP activity on repeats, these regions contain 45% to 68% of the repeat elements identified by RepeatMasker and are almost completely devoid of genes (from zero to 360 genes across all AT-rich regions, depending on the genome). There is 2-fold variation in the number of AT-rich blocks per genome (Table 2), with some species exhibiting a large number of relatively small blocks (e.g., *E. bromicola* has 808 blocks of average size 27.8 Kb), while other species have a small number of larger blocks (e.g., *E. amarillans* has only 351 blocks but an average size of 44.4 Kb).

### 3.3. Chromosome Structure across the Genus Is Highly Rearranged despite Conservation of Chromosome Number

To assess chromosome evolution across the genus, we first looked at large-scale rearrangements of coding regions through synteny analyses using MCScan. We found that rearrangements of coding regions both within and between chromosomes are extremely common (Figure 1B,C). Indeed, the degree of restructuring is so substantial that it prevents numbering of chromosomes on homology grounds, so we instead number chromosomes from largest to smallest within each species. As expected, species in basal positions in the phylogeny (*E. gansuensis*, *E. glyceriae*) have the most rearrangements relative to other *Epichloë* genomes (Figure 1B), but variation is even observed between strains within the same species (Figure 1C). One of the most striking patterns from these data is the strict conservation of chromosome number despite the high degree of rearrangement. Therefore, there is a surprising lack of association between the extent of rearrangements and chromosome number in *Epichloë*.

The extensive nature of the chromosomal rearrangements prompted us to examine patterns of rearrangements at different evolutionary distances at the single chromosome level. Taking sequences homologous to *E. clarkii* chromosome 2 as an exemplar, shifts of chromosomal content from one chromosome to another occurred four times in the evolutionary history of the genus (Figure 1C). In the basal species *E. gansuensis* and *E. scottii*, content homologous to this chromosome was located on two distinct chromosomes (chromosomes 1 and 6 in *E. gansuensis* and chromosomes 1 and 4 in *E. scottii*), with minor rearrangements between the two taxa. Consolidation into a single chromosome occurred on the branch linking *E. gansuensis* and *E. scottii* to the rest of the genus. Once formed, this configuration persisted in most derived species, including the *typhina* species complex (*E. clarkii*, *E. poae* and both strains of *E. typhina*), the *E. bromicola* and *E. elymi* monophyletic clade, and the single species lineages of *E. glyceriae* and *E. brachyelytri*. Only minor internal chromosomal rearrangements differentiate this chromosome in these seven species. However, two further chromosome rearrangements occurred in the clade comprising *E. amarillans*, *E. baconii* and *E. festucae*, distributing the homologous sequence content across two chromosomes (chromosome 1 and 6 in *E. festucae*, chromosome 3 and 5 in *E. amarillans*) or three chromosomes (chromosome 1, 5 and 6 in *E. baconii*).

### 3.4. Chromosome Rearrangements Are Not Correlated with Nucleotide Scale Changes

Unlike gene based synteny analyses, whole genome alignments can identify and quantify sequence rearrangements that occur at small scales and in non-coding regions. To investigate the patterns of variation from single nucleotide to large sequence rearrangements across the genus, we performed a reference-free whole genome sequence alignment using the framework provided by Progressive Cactus. This method uses ancestral sequence reconstruction in its alignment process, which allows us to quantify mutations and sequence rearrangements occurring between internal nodes of the tree and extant species, providing a framework to quantify changes across different evolutionary distances. Ancestral genomes reconstructed at internal nodes of the *Epichloë* phylogeny each constituted an assemblage of 269 to 1538 fragmented sequences. As such, they do not capture the chromosomal-level configuration of ancestral genomes and only allow localized reconstructions of ancient sequences. Quantified on the terminal branches of the tree between extant and closest ancestral genomes, substitutions were the most common class of mutation, with up to 1,497,975 substitutions on the longest branch of the tree. Insertions were more common than deletions, and inversions were the rarest class of mutation (Figure 2C, Table 3). Unsurprisingly, across the entire genome (both coding and non-coding regions), transitions, transversions and substitutions as a whole correlated most with the evolutionary distances inferred from the single copy ortholog genes (Figure 2B; R^2^ = 0.81 to 0.89, all *p* < 1 × 10^−5^). Insertions and deletions exhibited more moderate correlations (R^2^ = 0.57 and 0.68, *p* = 6.6 × 10^−4^ and 9.4 × 10^−5^). Most strikingly, however, rearrangements did not correlate with evolutionary distances (R^2^ = 0 to 0.006, *p* = 0.32 to 0.95). These results suggest the surprising conclusion that rearrangements in *Epichloë* have separate evolutionary dynamics to nucleotide-level mutational changes.

It has previously been suggested that transposable elements and AT-rich repeat regions facilitate chromosomal rearrangements [36]. Therefore, we looked to see the extent to which the chromosomal rearrangements we observe in *Epichloë* genomes are associated with AT-rich regions. We found that AT-rich regions were most impacted by rearrangements, with changes between ancestral and extant genomes affecting between 43% and 71% of all nucleotides within these regions (Figure 2D). In contrast, rearrangements only affected 1 to 11% of the nucleotides in GC-rich regions. Overall, genomes with larger AT-rich regions were also found to have undergone larger numbers of sequence rearrangements (R^2^ = 0.46, *p* = 0.006 for the correlation between the size of the AT-rich regions and the total number of observed rearrangements). These results indicate that most of the chromosomal level structural changes observed in *Epichloë* have been driven by recombination breakages in repeat elements, which in turn suggests that these recombination events (or at least the fixation of them) and nucleotide-level mutations have had different dynamics during the course of *Epichloë* evolution.

## 4. Discussion

Most studies of genome structure have focused either on variation within a species [37,38] or across widely divergent taxa [39]. Here we report complete telomere-to-telomere genomes for 15 strains in 12 species within the *Epichloë* genus, which includes representatives of all the major clades in this globally distributed fungal endophyte group. These genomes show ~1.4-fold variation in size (33.2–46.2 Mb), with the majority of this variation being driven by changes in genomic repeat content, particularly transposable elements, as has been observed for many other species across kingdoms [40,41]. The completeness of these genomes enables detailed comparisons of their genome evolution over both long and short timeframes.

One of the most striking observations from this study is the chromosome number conservatism of *Epichloë*. Earlier attempts to resolve ploidy using pulsed-field gel electrophoresis raised the possibility of variation in chromosome number within the genus. However, based on the data presented here, all non-hybrid *Epichloë* species studied so far have exactly seven nuclear chromosomes. This absolute stasis in chromosome number has not been observed in other fungi genera as far as we are aware. The most interesting aspect of this observation is that it comes despite extensive rearrangements within and between these chromosomes. A possible explanation for the constant chromosome number, despite extensive rearrangement, is constrained expression of the proteins involved in binding to centromere regions. Centromeres determine chromosome number and are defined epigenetically in *Epichloë*, not by centromere-specific repeats [16], so centromere number may be determined by the expression level of these molecular components irrespective of how much rearrangement has occurred. Alternatively, *Epichloë* may be particularly recalcitrant to *de novo* centromere formation/loss, thus rendering all di/acentric chromosomes lethal. The fact that some *Epichloë* allopolyploids appear to have a chromosome number that is not a multiple of seven [42] may provide an avenue to test these hypotheses experimentally.

The most surprising observation in this study is that the numbers of chromosomal level rearrangements are uncorrelated with nucleotide-level mutations in *Epichloë*. We show that nucleotide-base mutations accumulate gradually over evolutionary time with some variation between species, much as expected (Figure 2B). However, detailed reconstruction across the genus reveals that some genomes rearrange rapidly over short time spans, while others have changed little over much deeper evolutionary time. Although chronological timelines are not known with great precision for *Epichloë* [43], some chromosome structures, such as the conserved chromosome formation shown in Figure 1C between the evolutionarily distant *E. brachyelytri* and *E. elymi*, appear to have persisted largely unchanged over the course of millions of years [44]. In contrast, other chromosomes are punctuated by bursts of rapid change, with major rearrangements having occurred over relatively short evolutionary timeframes, such as the same chromosome that shows deep evolutionary stasis between *E. brachyelytri* and *E. elymi* being substantially restructured in the monophyletic clade comprising *E. festucae*, *E. amarillans* and *E. baconii*. Thus, the disconnect between the rates of nucleotide-level changes and rearrangements appears to lie primarily with discontinuous rearrangement rates.

What is the cause of the periods of relative rearrangement stasis versus punctuated change? Our data show that most rearrangements occur in the AT-rich repeat regions (Figure 2D), implicating these regions in the rearrangements. As transposons are numerous in AT-rich regions, albeit often deactivated by RIP mutations, the rearrangements may be a consequence of transposon-mediated recombination events [45,46] that are induced by transposon mobility and/or the multiple homology targets that are provided by transposon copies. AT-rich regions differ substantially between *Epichloë* strains, thereby suggesting that these differences might underlie the different rearrangement rates.

An alternative explanation is that the periodic nature of rearrangements simply reflects stochastic occurrence. Chromosomal rearrangements tend to be neutral as long as they do not disrupt the function of essential genes or the capacity for sexual crossing. Many rearrangements may therefore be fortuitous, rare events that became fixed within populations through the standard processes of random genetic drift. The absolute number of rearrangements is far smaller than the number of nucleotide-level changes, thus also favoring the idea that many rearrangements may be chance events.

A third, not mutually exclusive, explanation is that some rearrangements were beneficial, and thus have been fixed by selection. In this case, the discontinuous occurrence simply reflects the stochastic nature of beneficial rearrangements arising. Chromosomal rearrangements alter 3D associations of DNA in the nucleus, as well as methylation and transcription of genes near break points, thus providing possible beneficial effects [47]. In addition, proximity to repeat elements affects the expression of genes in *Epichloë* [16], so repositioning of genes through chromosomal rearrangement might lead to altered functional outcomes. It would be interesting to determine whether the expression levels of genes near the break points identified in the synteny analysis, particular those between closely related strains such as *E. festucae* Fl1 and E437, have significant fitness consequences. It is also possible that chromosome rearrangements have driven speciation, since large genomic rearrangements are thought to be a cause of reproductive isolation. Consistent with this, rearrangements have been found to correlate with species richness across fungi phylogenetic groups [48]. However, while we find major rearrangements at the origin of some clades, other clades share no common chromosome restructurings, and chromosome structure variation sometimes occurs even between strains within species. Therefore, the rearrangements we observe in *Epichloë* are unlikely to be solely a consequence of their effects on speciation.

## 5. Conclusions

To better understand the evolution of fungal chromosome structure, we present 15 complete telomere-to-telomere genomes from 12 *Epichloë* species. The key trend we observe, a striking dichotomy between periods of stability and change, was unexpected. While some aspects of chromosome structure, such as the constrained number of chromosomes, appears to be surprisingly conservative, other aspects such as intra- and inter-chromosomal reordering can occur remarkably quickly. The punctuated nature of this genome-scale evolutionary change, and its underlying causes, is an obvious question for future work, which this set of complete genomes across an entire genus will help enable.

## Figures and Tables

**Table 1:** Summary information for the 15 *Epichloë* strains.

Species	Strain	Sequencing Technology	Host Species	Geographical Origin	Sexual Reproduction	NCBI BioProject Number
*E. amarillans*	NFe708	PacBio RSII/Illumina	*Elymus canadensis*	Iowa, USA	Observed	PRJNA532945
*E. baconii*	E357	MinION	*Calamagrostis villosa*	Switzerland	Observed	PRJNA842087
*E. brachyelytri*	E1124	MinION	*Brachyelytrum erectum*	New York, USA	Observed	PRJNA841758
*E. bromicola*	NFe1	MinION/Illumina	*Hordeum bogdanii*	Kazakhstan	Not observed	PRJNA842763
*E. clarkii*	1605_22	PacBio RSII/Illumina	*Holcus lanatus*	Switzerland	Observed	PRJNA533212
*E. elymi*	NFe728	MinION	*Elymus virginicus*	Oklahoma, USA	Observed	PRJNA839151
*E. elymi*	NFe732	PacBio RSII/Illumina	*Elymus canadensis*	Oklahoma, USA	Not observed	PRJNA532946
*E. festucae*	E437	PacBio RSII/Illumina	*Festuca pulchella*	Japan	Observed	PRJNA842088
*E. festucae*	Fl1	PacBio RSII/Illumina	*Festuca longifolia*	United Kingdom	Observed	PRJNA431450
*E. gansuensis*	e7080	MinION	*Achnatherum inebrians*	China	Not observed	PRJNA842754
*E. glyceriae*	E2772	MinION	*Glyceria striata*	New York, USA	Observed	PRJNA841696
*E. poae*	Pn1703_3	PacBio Sequel II/Illumina	*Poa nemoralis*	France	Observed	PRJNA843480
*E. scottii*	DSM112488	MinION	*Melica uniflora*	Germany	Observed	PRJNA756890
*E. typhina*	Pt1807_3	PacBio Sequel II/Illumina	*Poa trivialis*	Spain	Observed	PRJNA844123
*E. typhina*	1756	PacBio RSII/Illumina	*Dactylis glomerata*	Switzerland	Observed	PRJNA533210

**Table 2:** Genomic characteristics of the 15 *Epichloë* strains.

Species	Strain	Chromosomes	Genome Length	Number of Genes	Repeat Regions (%)	AT-Rich Regions (%)	Number of AT-Rich Blocks	Mean Length of AT-Rich Blocks
*E. amarillans*	NFe708	7	38,224,169	7451	68	41	351	44,429
*E. baconii*	E357	7	39,250,507	7486	67	39	528	29,265
*E. brachyelytri*	E1124	7	44,747,857	7335	72	49	777	28,132
*E. bromicola*	NFe1	7	46,201,037	7615	72	49	808	27,892
*E. clarkii*	1605_22	7	45,646,793	7772	72	49	650	34,135
*E. elymi*	NFe728	7	34,206,040	7424	64	32	340	32,533
*E. elymi*	NFe732	7	33,820,330	8324	63	32	348	30,920
*E. festucae*	E437	7	33,219,473	7764	61	28	395	23,630
*E. festucae*	Fl1	7	35,023,690	7822	63	32	435	25,714
*E. gansuensis*	e7080	7	39,568,882	7601	63	37	428	33,994
*E. glyceriae*	E2772	7	43,207,551	7267	71	47	496	40,681
*E. poae*	Pn1703_3	7	34,185,484	7420	63	32	396	27,983
*E. scottii*	DSM112488	7	37,342,247	6486	62	37	363	29,549
*E. typhina*	Pt1807_3	7	39,303,283	7305	68	41	513	31,484
*E. typhina*	1756	7	33,870,766	7539	64	32	503	27,726

**Table 3:** Numbers of each mutation class accumulated on terminal branches of the *Epichloë* phylogeny, between ancestral and extant genomes.

Genome	Terminal Branch Length	Substitutions	Transitions	Transversions	Insertions	Deletions	Inversions	Duplications	Transpositions
*E. amarillans*	0.009439	413,037	278,990	134,047	7030	2924	0	16,594	137
*E. baconii*	0.01839	608,309	320,946	287,363	10,798	962	1	14,582	50
*E. brachyelytri*	0.034203	772,918	465,715	307,203	11,648	3,713	0	53,611	193
*E. bromicola*	0.016056	794,167	464,710	329,457	13,291	2,241	2	20,666	59
*E. clarkii*	0.006319	493,578	296,234	197,344	14,452	1,137	1	60,699	312
*E. festucae* (E437)	0.001888	307,728	173,466	134,262	6016	421	1	13,191	102
*E. festucae* (Fl1)	0.001466	234,349	139,930	94,419	7410	403	1	29,260	172
*E. gansuensis*	0.055092	1,497,975	921,662	576,313	20,821	4835	1	25,934	116
*E. glyceriae*	0.023931	710,405	475,456	234,949	7697	4900	0	27,682	166
*E. elymi* (NFe728)	0.000835	81,944	55,421	26,523	3946	243	0	5937	68
*E. elymi* (NFe732)	0.000892	156,109	99,732	56,377	3891	248	3	6421	80
*E. poae*	0.009131	500,740	283,538	217,202	7292	1025	0	11,467	46
*E. scottii*	0.024456	649,936	408,925	241,011	11,654	4056	3	25,089	166
*E. typhina* (Pt1807)	0.010108	594,494	353,680	240,814	11,336	2080	1	42,243	242
*E. typhina* (1756)	0.009271	455,536	264,745	190,791	9370	932	1	24,706	202

## Data Availability

Data underlying the analyses presented here have been deposited at NCBI under the BioProject numbers listed in Table 1.
